# Embryo and Its Mitochondria

**DOI:** 10.3390/antiox10020139

**Published:** 2021-01-20

**Authors:** Pascale May-Panloup, Magalie Boguenet, Hady El Hachem, Pierre-Emmanuel Bouet, Pascal Reynier

**Affiliations:** 1 Reproductive Biology Unit, Angers University Hospital, 49000 Angers, France; 2 MITOVASC, Angers University, INSERM 1083-CNRS 6015, IBS–CHU, 49000 Angers, France; magalie.boguenet@univ-angers.fr (M.B.); pareynier@chu-angers.fr (P.R.); 3 Department of Reproductive Medicine, Saint Joseph Fertility Center, Beirut 1100, Lebanon; hadyelhachem@gmail.com; 4 Department of Reproductive Medicine, Angers University Hospital, 49000 Angers, France; PierreEmmanuel.Bouet@chu-angers.fr; 5 Department of Biochemistry and Genetics, Angers University Hospital, 49000 Angers, France

**Keywords:** embryo, mitochondria, mitochondrial DNA

## Abstract

The mitochondria, present in almost all eukaryotic cells, produce energy but also contribute to many other essential cellular functions. One of the unique characteristics of the mitochondria is that they have their own genome, which is only maternally transmitted via highly specific mechanisms that occur during gametogenesis and embryogenesis. The mature oocyte has the highest mitochondrial DNA copy number of any cell. This high mitochondrial mass is directly correlated to the capacity of the oocyte to support the early stages of embryo development in many species. Indeed, the subtle energetic and metabolic modifications that are necessary for each of the key steps of early embryonic development rely heavily on the oocyte’s mitochondrial load and activity. For example, epigenetic reprogramming depends on the metabolic cofactors produced by the mitochondrial metabolism, and the reactive oxygen species derived from the mitochondrial respiratory chain are essential for the regulation of cell signaling in the embryo. All these elements have also led scientists to consider the mitochondria as a potential biomarker of oocyte competence and embryo viability, as well as a key target for future potential therapies. However, more studies are needed to confirm these findings. This review article summarizes the past two decades of research that have led to the current understanding of mitochondrial functions in reproduction

## 1. Introduction

**Figure 1:**
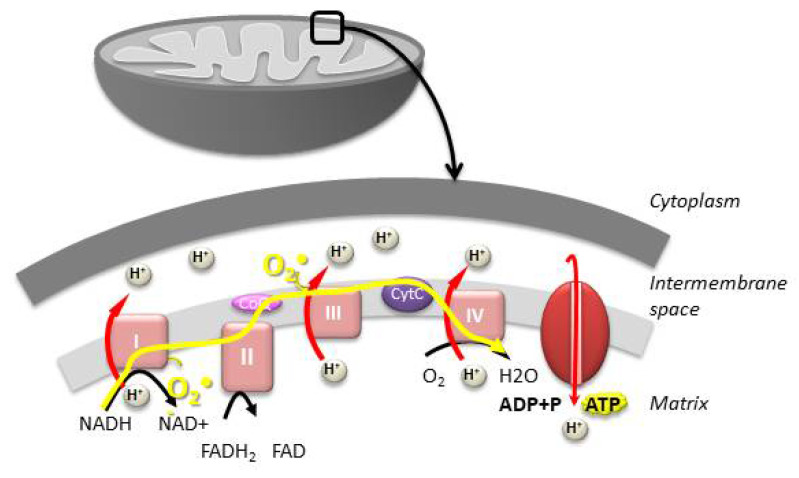
The mitochondria play a central role in energy production by ATP synthesis via oxidative phosphorylation (OXPHOS). OXPHOS uses four large multi-enzymatic complexes (complexes I to IV), which constitute the electron transport chain, and complex V, which synthesizes ATP. OXPHOS activity generates the vast majority of reactive oxygen species (ROS) (O_2_^●−^).

The mitochondria are small organelles found in all eukaryotic cells and are thought to have evolved from the endosymbiosis between an ancestral eukaryotic cell and an α-proteobacteria capable of metabolizing oxygen [1]. They are responsible for the production of more than 90% of the ATP necessary for cellular function via oxidative phosphorylation (OXPHOS) (Figure 1). OXPHOS uses five large multi-enzymatic complexes found in the cristae of the inner mitochondrial membrane: complexes I to IV that constitute the electron transport chain (ETC), and complex V (ATP synthase), which allows synthesizing ATP by phosphorylation using the energy generated by the translocation of protons across the inner membrane. OXPHOS functioning produces most of the endogenous reactive oxygen species (ROS), which are implicated in many cellular regulation pathways but could become toxic when they accumulate. Besides their crucial role in energy production, mitochondria play an essential role in the biosynthesis of organic compounds, apoptosis, calcium homeostasis, and thermogenesis, as well as cellular signal pathways and gene expression [2,3,4].

Each somatic cell contains hundreds of mitochondria that form a dynamic network capable of movement, fusion, and fission, depending on the cellular energy requirements [5]. These mitochondrial dynamics are mediated by GTPases (dynamin-related protein 1 (DRP1), optic atrophy protein 1 (OPA1), and mitofusins 1 and 2 (MFN1 and MFN2)) found in the mitochondrial membrane. Because of this network, it is often difficult to determine the total number of mitochondria, and the mitochondrial mass is indirectly estimated by quantifying the mitochondrial DNA (mtDNA). Indeed, mitochondria have their own genome, a small (about 16 Kilobases) circular double-stranded DNA [6]. MtDNA contains mostly coding regions that encode 13 subunits of the complexes involved in the respiratory chain, 2 rRNAs, and 22 tRNAs. The other mitochondrial proteins, estimated to be around 1500, are coded by the nuclear genome and later imported into the mitochondria.

The regulation of mitochondrial biogenesis is central to mitochondrial functions. It is engineered, in coordination with the general cell metabolism, by PGC1α (PPAR gamma coactivator 1) and NAD-dependent deacetylases of the sirtuin family. PGC1α is a transcription coregulator that controls the activity of transcription factors that activate nuclear genes involved in β oxidation, antioxidant defense, and the citric acid cycle (Krebs cycle). It also promotes the expression of NRF 1 and 2 (nuclear respiratory factors 1 and 2), which are transcription factors for nuclear genes coding for proteins involved in mitochondrial import, in assembly, and complexes of the respiratory chain, as well as factors involved in translation (tRNA and rRNA), transcription, and replication of the mtDNA, such as TFAM (mitochondrial transcription factor A) and POLG (polymerase gamma), thus allowing the coordination between the nuclear and mitochondrial genomes [7,8].

## 2. Mitochondria, Gametogenesis, and mtDNA Transmission

**Figure 2:**
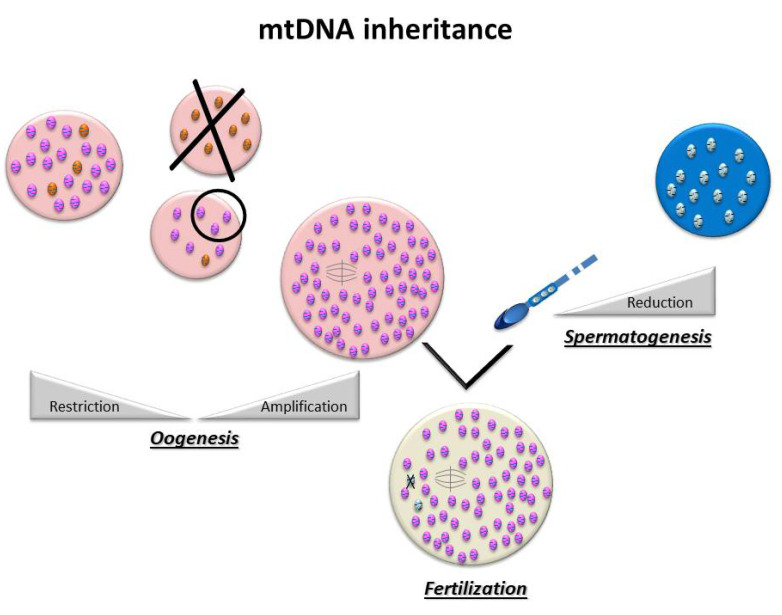
MtDNA transmission is uniparental: spermatozoa contain very few mitochondria that will be destroyed after fertilization, while in the oocyte, there is a global amplification of the mitochondrial pool that makes it the cell with the highest number of mitochondria in the organism.

For most organisms, maternal uniparental inheritance is the norm, as the mitochondrial genome is inherited exclusively from the oocyte (Figure 2) [9]. This is the consequence of two major events: first of all, paternal mtDNA is eliminated via several mechanisms: a drastic decrease in the number of mitochondria during spermatogenesis and an active destruction of mitochondria and/or paternal mtDNA during early embryogenesis [10,11]. On the other hand, there is global amplification of the mitochondrial pool during oogenesis, producing several hundred thousands of copies of mtDNA, making the mature oocyte the cell with the highest number of mitochondria in the organism, in all species [12]. In the human oocyte, the mean number of copies of mtDNA has been estimated to be around 250,000 [13].

Studies on mtDNA segregation in animal and human pedigrees affected by mtDNA mutations have shown that, when point mutations occur, there may be a high-level switching of mutants within a single generation [14,15,16,17,18]. This concept has led to the bottleneck theory [19], according to which a very small number of mtDNAs may populate the oocyte and, consequently, the organism. Based on this hypothesis, there is a significant decrease in the amount of mtDNA in the primordial germinal cells with very few founding mitochondria, followed by a notable amplification that occurs during oogenesis [20,21]. This random dispersal of a limited number of mitochondrial DNA is thought to be associated with other mechanisms [22], such as a preferential replication of a limited sub-population of mtDNA [23] or compartmentalization of these mtDNA molecules [24]. These phenomena would help in eliminating abnormal mitochondrial genomes and homogenizing mtDNA, thus preserving mitochondrial integrity across generations. Indeed, mtDNA has a mutation rate a hundred times higher than nuclear DNA [25]. This high rate is linked not only to replication errors but also to damages caused by ROS, mainly because of the proximity of mtDNA to the respiratory chain [26]. This observation, along with the quasi-absence of genome recombination [27], can explain the risk of accumulation of deleterious mutations over the years (Mueller’s rachet) [28].

The Bottleneck hypothesis can explain how mtDNA can be “refreshed” and “purified” from one generation to the next [27,29,30]. However, this phenomenon remains quite complex, as it varies considerably depending on the type of mtDNA variants and the nuclear background [31]. Indeed, several segregation models transmitting pathogenic mutations of mtDNA have been described in human lineages [32,33].

## 3. Mitochondria and the Embryo: Pathophysiological Aspects

### 3.1. The Importance of the Mitochondrial Pool for the Initiation of Embryo Development

Embryogenesis is rooted in oogenesis, and notably on the oocyte mitochondrial pool, which represents 30% of the oocyte volume and is essential for the initiation of embryo development [34]. Indeed, a low oocyte mtDNA content has been associated with fertilization failure [9] and abnormal embryo developments in humans [35,36,37] and other species [38,39].

### 3.2. Ensure a Sufficient Energy Production

**Figure 3:**
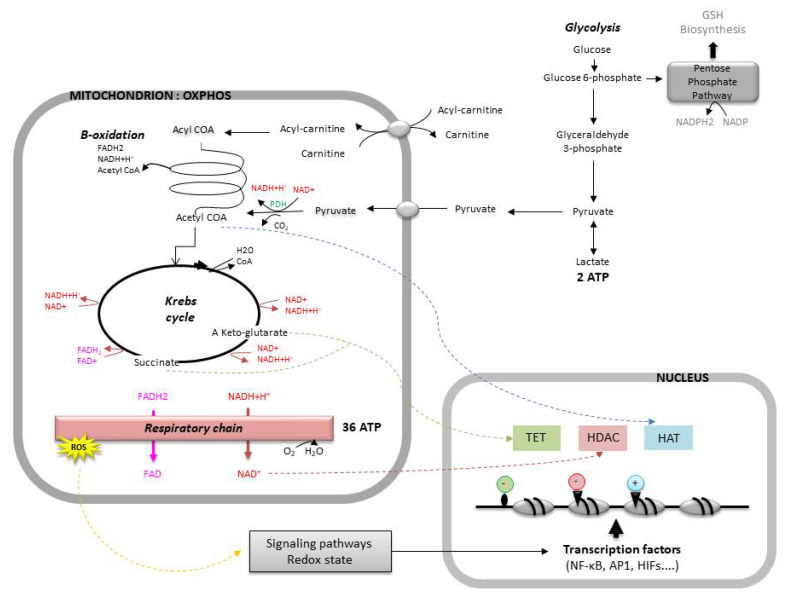
The role of mitochondria in cell metabolism, signaling pathways, and genic expression. The cell produces its energy (ATP) via oxidative phosphorylation (OXPHOS) or via anaerobic glycolysis. The embryo uses pyruvate or glucose as the main source of energy according to its stage of development. The mitochondrial function produces intermediate metabolites and reactive oxygen species (ROS) that are involved in signaling pathways, redox homeostasis, and genic expression. Enzymes implicated in epigenetic regulation: Ten eleven translocase (TET), histone deacetylase (HDAC), and histone acetyltransferase (HAT). Transcription factors: κB family (NF-κB), activator protein-1 family (AP1), and hypoxia-inducible transcription factors (HIFs). GSH, glutathione; PDH, pyruvate dehydrogenase.

As shown in several species, (mouse [40]; bovine [41]; rat [42]; pig [43] and human [44]) mtDNA does not increase during early embryogenesis. Indeed, except for a very brief period of turnover before the 2-cell stage, shown in mice [45] and pigs [46], mtDNA does not replicate until the blastocyst stage. During that time, the embryo metabolism depends mostly on pyruvate (and fatty acids in some species) via oxidative phosphorylation [47,48,49] (Figure 3). The mitochondrial mass in the oocyte must therefore be sufficient to be divided between the different blastomeres and allow them to have their own mitochondria producing enough ATP and metabolites essential to their functioning and development until mitochondrial biogenesis starts again. Therefore, an abnormal functioning of oocyte mitochondria, leading to a decrease in OXPHOS, can cause abnormal embryo development in humans [50,51]. Studies have linked insufficient ATP content with fertilization failure and abnormal embryo development [52]. Others have shown a difference in the ATP content between the blastomeres of an embryo related to a difference in the number of mitochondria [53]. They reported that, in humans, a defect in the distribution of mitochondria at the zygote stage could lead to a disproportionate inheritance between the blastomeres, which can lead to an insufficient ATP production and an inability to pursue division in the blastomeres with a low mtDNA copy number [53].

### 3.3. The Paradox of the Cleavage-Stage Embryo: Oxidative Metabolism and Low Mitochondrial Activity

The metabolisms of the oocyte and the cleavage stage embryo are oxidative, even though their mitochondria are poorly differentiated. Indeed, their mitochondria are spherical, measure less than 1 μm, have few cristae, have a low oxygen consumption and low ATP production [34,52,54]. Therefore, the global mitochondrial mass seems to be critical for the production of the energy needed by the embryo.

On the other hand, the mitochondria have the ability to position themselves in the cytoplasmic regions with a higher energetic demand, allowing them to support the oocyte maturation and embryo development [55,56,57]. The spatial organization of organelles, especially the mitochondria with a high membrane potential (Delta Ψ, reflecting their activity), has been correlated to the embryo development potential [51,58]. However, studies have shown significant variability in the distribution pattern of mitochondria during oogenesis and embryogenesis, most notably in mice, as well as significant differences between species [59].

The balance between the energy need and production could be related to the intricate relationship between mitochondrial activity and calcium signaling since calcium is an activator of OXPHOS, via activation of the dehydrogenases of the Krebs cycle, the respiratory chain, and ATP synthase. There are several contact zones between the endoplasmic reticulum (ER) and the mitochondria that allow the transmission of calcium signals between the two organelles, called the MAMs (mitochondria-associated membranes) [60]. This mechanism is known to play a role during fertilization when mitochondrial activation by the calcium released by the ER induces the calcium oscillations necessary for the activation of the oocyte [61].

Another possible explanation of the low oxygen consumption could be the use of the “adenosine salvage pathway”, described in the bovine oocyte, as an alternate method to produce ATP and meet the energy demands [62]. The adenosine salvage pathway is a two-step enzymatic reaction that phosphorylates AMP to ADP via the adenylate cyclase and the ADP to ATP via the creatine kinase.

It is worth noting that the relocation of the mitochondria with low ATP production during embryogenesis to certain specific areas could be intended to allow them to fulfill, in these specific areas (most notably perinuclear), functions other than energy production [63,64].

#### 3.3.1. Limiting Oxidative Stress

Maintaining a low OXPHOS activity, stimulated only locally if necessary [56,57], would allow limiting the oxidative stress. Indeed, an increase in ATP demand causes an increase in the OXPHOS activity. The respiratory chain transfers electrons to molecular oxygen, which is reduced to water. However, the leakage of respiratory chain electrons during ETC functioning leads, in 1 to 3% of cases, to the formation of superoxide (O_2_^●−^). More than 95% of ROS are generated by the respiratory chain [65]. O_2_^●−^ is generated in several mitochondrial sites, especially by complexes I, II, and III of the respiratory chain [66]. Complexes I and II exclusively provide O_2_^●−^ in the mitochondrial matrix, while complex III produces O_2_^●−^ in both the matrix and intermembrane space [67]. The O_2_^●−^ generated in the matrix is converted to H_2_O_2_ by mitochondrial superoxide dismutase protein 2 (SOD2) [68]. The O_2_^●−^ released in the intermembrane space joins the cytosol, through voltage-dependent anion channels in the outer mitochondrial membrane, and can be converted into H_2_O_2_ by cytosolic superoxide dismutase 1 (SOD1) [69].

ROS act as a signal of mitochondrial dysfunction and play a role in triggering the cellular repair mechanisms and apoptosis. When they accumulate at supraphysiologic levels, and because of their high chemical reactivity, they can modify different biomolecules such as lipids, proteins, and nucleic acids and cause molecular and cellular damage [70,71].

In vitro exposure of mammal embryos to oxidative stress has been linked to mitochondrial dysfunction, DNA lesions, embryo development arrest, and death (mouse [72,73]; bovine [74,75]; human [76]). In humans, studies have shown that culture conditions, especially culture under atmospheric oxygen concentration, can generate oxidative stress, as reflected by increased ROS, modification of embryo metabolism, and DNA damages [76]. This observation is further supported by the fact that human embryo development is improved at a reduced oxygen concentration of 5% [77,78,79,80].

In mice, when compared to culture under physiologic conditions (5% O_2_), culture under atmospheric conditions (20% O_2_) leads to increased ROS levels in the embryo, morphologic and functional mitochondrial anomalies, and a modification of the expression profile of several mitochondrial genes [81]. On the other hand, in vitro animal studies have shown that the regulation of the reducing environment, by using anti-free radicals (antioxidants) or reducing agents, could improve the development of preimplantation embryos [82].

Finally, mtDNA is particularly vulnerable to ROS, given its proximity to the respiratory chain and its lack of protective histones and efficient repair mechanisms. So mtDNA is located in a potentially mutagenic hostile environment, which can compromise the integrity of the genetic information transmitted to the offspring.

Thus, regulated ROS levels are crucial to avoid oxidative damage in the embryo. This regulation allows maintaining the balance between pro and antioxidant agents, which defines the redox status of the embryo.

#### 3.3.2. Maintaining Redox Homeostasis

The cellular redox status has a significant impact on several cellular functions, such as energy supply, proliferation, differentiation, and apoptosis [83]. The control of the redox equilibrium, and particularly the production of ROS at physiologic levels, is crucial for embryo physiology [84]. Indeed, animal models have shown that an increase in the ROS levels right after fertilization was associated with better embryo development, thus suggesting a possible interaction between redox regulation, mitochondrial metabolism, and calcium oscillations [85] (Bovine). On the other hand, brief exposure of bovine embryos to H_2_O_2_ during embryo genome activation (EGA) promotes embryo development [86]. These mechanisms are not well defined in humans.

ROS are known to act as signaling molecules and contribute to signaling pathways that control different cellular processes [87]. Indeed, studies have shown that H_2_O_2_ generated by complex III and released into the cytoplasm can play a part in cell signaling pathways [88]. It acts via the oxidation of the cysteine residues of redox-sensitive proteins, thus modifying their conformation and activity, and causing remodeling of the signal transduction, which directly impacts several signalization cascades [89,90]. H_2_O_2_ has the capacity to affect the signaling pathways of growth factors that are important for cellular growth and proliferation, with one of the most important mechanisms being the inactivation of the protein tyrosine phosphatases (PTPs). PTPs downregulate these signaling pathways and have, in their catalytic domain, cysteine residues that can be inactivated by oxidation by H_2_O_2_ [91]. One of these PTPs, phosphatase and tensin homolog (PTEN) [92], when inactivated, leads to the activation of Akt, a regulator of the PI3K pathway that stimulates proliferation [93]. Another one is Cdc14B, whose inactivation leads to the activation of the cyclin-dependent kinase 1 (Cdk1), which promotes mitotic progression [94]. Other phosphatases, such as the mitogen-activated protein kinase (MAPK), are also similarly impacted [91]. On the other hand, ROS can directly target several kinases (Src family kinases) [95,96] and transcription factors (activator protein AP1) [97].

Among the several transcription factors impacted by the alteration of the redox homeostasis, some play an important role in embryogenesis (Figure 3). For instance, the expression of members of the nuclear factor κB family (NF-κB), which activate the expression of many genes (cytokines, growth factors, adhesion molecules, enzymes of the redox system, etc.), depends on the ROS levels. A slight increase in the ROS levels activates NF-κB, whereas an excess decreases its capacity to bind to DNA [98,99]. Likewise, proteins of the activator protein-1 family (AP1), which regulate the expression of several genes, as well as processes of cellular differentiation, proliferation, and apoptosis [100], are controlled by redox-dependent mechanisms. Other transcription factors, such as the nuclear factor erythroid-derived 2-related factors 1 and 2 (Nrd1/2) and the hypoxia-inducible transcription factors (HIFs), both of which will be covered later in this review, also depend on the redox status of the embryo [84].

On the other hand, the redox regulation in the embryo seems to be related to the regulation of the cellular cycle, via the activation of factors such as the M-phase promoting factor (MPF) and other CDK-cyclin complexes [101].

The energy requirements of the mammal embryo change over the course of its development. The embryo must be able to modify its metabolism and adapt to its environment. During tubal-uterine transport, the embryo goes through different environments with different compositions and conditions, and is exposed to a decreasing oxygen gradient while simultaneously shifting from an oxidative metabolism to a glycolytic one.

During this period, several metabolic factors will act as sensors of cellular and extracellular conditions, and accordingly, guide the metabolism and influence the development program. The mitochondrion is at the center of the regulation of the metabolic homeostasis of the embryo via the modulation, the disponibility of metabolites, and the redox status [102].

Indeed, mitochondria play a central role in the redox equilibrium of the embryo. This equilibrium depends on the ratio of several redox couples present in the cell, the most important being: reduced/oxidized glutathione (GSH/GSSG), reduced/oxidized nicotinamide adenine dinucleotide (NADH/NAD^+^), and reduced/oxidized nicotinamide adenine dinucleotide phosphate (NADPH/NADP^+^). The mitochondria regulate the NAD(P)H/NAD(P)^+^ ratios and produce ROS capable of unbalancing this redox status, as well as intermediate metabolites necessary for the regeneration of embryo antioxidants. The equilibrium of these redox couples is finely regulated during embryo development. The glutathione level increases after EGA and is progressively depleted until the blastocyst stage [103]. Moreover, increasing intracellular concentrations of reduced glutathione, especially during oocyte maturation, are associated with both improved fertilization and subsequent embryo development in vitro (pig [104]; bovine [105]).

The mitochondria allow the embryo to adapt to the environmental conditions it is exposed to. The embryo metabolic shift is the result of the balance between the expression of enzymes implicated in the energy metabolism, directly regulated by oxygen sensing, and the redox status of the embryo, all orchestrated by the mitochondria. The uterus, where the oxygen concentration is between 1.5 and 1.8%, constitutes a hypoxic environment for the embryo [106]. In these conditions, the mitochondria undergo morphological changes and paradoxically increase ROS production by complex III [107,108,109,110]. The ROS activate the HIF proteins by stabilizing the HIFα subunit. HIFs activity is influenced by Krebs cycle metabolites, most importantly, α-ketoglutarate [111]. Activated HIFs are transcription factors of genes that regulate the cellular adaptation to hypoxia. They activate a series of adaptive responses implicating the mitochondria. These proteins are capable of modulating the constitution and the activity of the respiratory chain by triggering the replacement of certain subunits of the complexes I to IV of the respiratory chain [112]. Therefore, hypoxia, by activating HIFs, modifies the functioning of the respiratory chain and allows to maintain ATP production while regulating the production of ROS [113,114]. A recent analysis of human embryos by microarray has confirmed the impact of the oxygen concentration on the expression of the genes of the metabolism, cell cycle, and OXPHOS [115]. These data confirm the intricate relationship between the environmental conditions (most notably O_2_ concentration), the mitochondria, and the redox status, as well as their influence on embryo development.

#### 3.3.3. Transcriptional Regulation and Embryo Genome Activation

A direct link between mitochondrial metabolic activity and chromatin dynamics has recently been reported. Indeed, the intermediate metabolites of the cellular metabolism are also cofactors of genetic reprogramming [2,64]. For instance, α-ketoglutarate and succinate, both byproducts of the Krebs cycle, regulate the activity of TET (ten-eleven translocase) proteins, a family of dioxygenases that promote DNA demethylation. Likewise, acetyl-coenzyme A provides the acetyl group required for the acetylation of histones by HATs (histone acetyl-transferases). Finally, NAD^+^ is capable of regulating the activity of HDAC (histone deacetylase) enzymes (Figure 3).

The most important epigenetic changes occur during the peri-implantation period, which makes it very sensitive to metabolic perturbations. A recent study found that many enzymes involved in the Krebs cycle are directly involved in the epigenetic remodeling that occurs during the EGA. According to the study, these enzymes are capable of partially and transiently relocating into the nucleus of the blastomeres, via a mechanism still to be identified (transport vesicles or chaperones proteins?). Once in the nucleus, these enzymes become active and provide the cofactors necessary for epigenetic regulation. In mice, blocking this system is correlated to the loss of the histones specific modifications and blocks the EGA. In humans, pyruvate dehydrogenase has also been found in the nucleus at the time of EGA at the 4/8-cell stage [116]. The short phase during which mtDNA replicates-a phase that has been confirmed in certain animal species [45,46] could therefore be crucial for embryo development. It would provide the mitochondrial pool needed for the production of intermediate metabolites and enzymes necessary for EGA.

In conclusion, at the cleavage stage, the role of mitochondria is not only to synthesize ATP, but also to maintain the redox homeostasis and produce intermediate metabolites that are essential to cell signaling pathways and genic expression [117]. Any mitochondrial dysfunction or excessive functioning can cause a perturbation in embryo development. This explains the importance of the mitochondrial pool and the disparity between pyruvate consumption and ATP production [117]. Thus, during this specific period, a low mitochondrial activity would favor a “quiet metabolism” or, more specifically, a balanced and parsimonious metabolism [118].

### 3.4. Resumption of Mitochondrial Biogenesis: From Morula to Blastocyst

**Figure 4:**
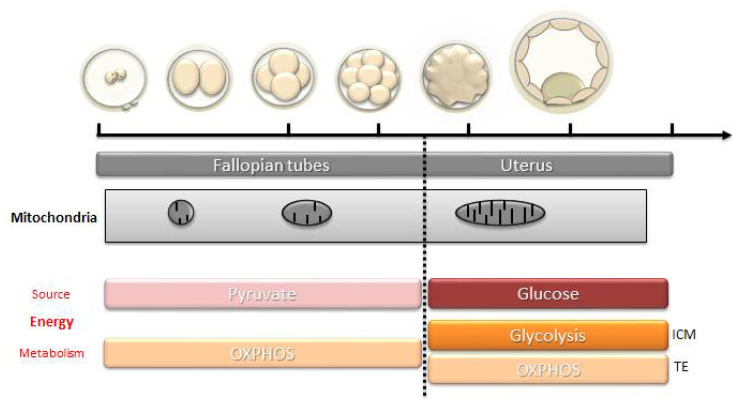
Mitochondrial modifications, energy source, and type of metabolism during the different stages of embryo development. ICM, inner cell mass, TE: trophectoderm.

The resumption of mitochondrial biogenesis occurs progressively (Figure 4). It corresponds to the resumption of mtDNA replication and the conversion of mitochondria into active forms (elongated form, development of mitochondrial cristae). In parallel, a significant increase in the consumption of glucose and oxygen is noted [119]. Mitochondrial biogenesis is maximal around the time of implantation, at the blastocyst stage, which marks the beginning of the differentiation of the embryo in large mammals [120], and at the stage of the egg cylinder in rodents [40]. At the blastocyst stage, the trophectoderm is the first to have a mitochondrial differentiation [121], which is essential for its own differentiation [122]. The energy needs are high to ensure the normal functioning of the Na/K ATPase pumps and the formation of the blastocele, with the cells using mainly glucose via the oxidative pathway. Meanwhile, the cells of the inner cell mass multiply, keep their pluripotent state and prepare the increase in the biomass of the embryo during the implantation in a hypoxic environment. These events in the inner cell mass depend on a glycolytic metabolism that favors the production of precursors for protein biosynthesis (glucose directed towards the pentose phosphate pathway that generates NADPH, which is essential for biosynthesis) and rapid cell divisions [123] (Figure 3).

#### 3.4.1. The Set-Point Theory

The oocyte mitochondrial pool is progressively divided between the different blastomeres of the embryo. For a given cellular lineage, the point with the lowest content in mtDNA constitutes the “set point” after which the mtDNA replication resumes, along with the cellular differentiation and the loss of pluripotency [124]. The resumption of the mitochondrial biogenesis would occur in parallel to the first differentiation events occurring at the morula stage in the trophectoderm. The cells of the inner call mass start their differentiation later, leading to the different cell lineages. Among these, the primordial germinal cells are the ones that will maintain the lowest level of mtDNA [124], and the mitochondrial biogenesis will only resume with the oocyte growth during folliculogenesis, leading to the formation of the mitochondrial pool of the mature oocyte.

#### 3.4.2. Abnormal Resumption of Mitochondrial Biogenesis

Some authors suggested that an insufficient oocyte mitochondrial pool, or an anomaly in the embryo, could trigger a compensatory mechanism that leads to a premature resumption of mitochondrial biogenesis [125]. A premature resumption of the biogenesis at a stage where the embryo’s metabolism is low could alter the balance between the demand and the mitochondrial activity and lead to oxidative damages and dysregulation of the cellular signaling pathways, which could negatively impact key steps of embryogenesis. Interestingly, in a recent study about early embryogenesis in mice, we reported a dysregulation of mitochondrial biogenesis that occurs with aging. It seemed that the absence of the replication burst that usually occurs at the 2-cell stage, along with the premature onset of the setpoint, could lead to an abnormal resumption of mtDNA replication and an inadequate timing of the differentiation events [126].

Overall, during early embryo development, the mitochondria undergo structural and positional changes that allow them to provide the energy needs of the embryo, while modulating the environment upon which depends nuclear epigenetic programming and the key events of preimplantation development [63].

## 4. Mitochondria and Biomarkers of Embryo Development

In the field of assisted reproductive technologies, the current research is focused on finding biomarkers for the implantation potential of embryos in order to improve the success rates (increase the pregnancy rates while reducing the multiple pregnancy rates). The mitochondria have been shown to be an essential part of oocyte quality and embryo development and have been at the center of many biomarkers studies.

### 4.1. Mitochondria of the Cumulus Cells

In the ovarian follicle, the oocyte is surrounded by cumulus granulosa cells (CCs), which are intimately involved in oocyte growth and maturation. Thus, oocyte competence is acquired through bidirectional signaling between the oocyte and the surrounding CCs [127,128]. In particular, oocyte-cumulus complex (OCC) interactions orchestrate carbohydrate, lipid, and protein metabolisms to provide the appropriate balance of energy required for the oocyte to undergo meiosis and fertilization and to support early embryogenesis [129,130,131]. Thus, CCs metabolize glucose from the blood circulation into pyruvate, which is then provided to the oocyte to allow ATP production by OXPHOS [132]. In turn, the oocyte regulates glycolysis in CCs by inducing the expression of glycolytic key genes [133,134,135]. Similarly, within the OCC, fatty acid β-oxidation from lipid produces additional ATP for meiotic resumption [136,137,138] and amino acid turnover [135,139] to ensure the metabolic needs of the oocyte. Granulosa cells and oocyte mitochondria, central agents of these metabolic pathways, are thus directly involved in establishing oocyte competence to support embryogenesis. Interestingly, it seems that CCs could regulate their mtDNA levels by mechanisms of degradation/replication in order to cater to the energetic needs of the oocyte they surround [140].

In this context, several authors have postulated that the mitochondria of the CCs could reflect the oocyte competence and that their analysis could offer the best non-invasive method to assess the metabolic events inherently related to the oocyte quality. Indeed, one study found that oocyte mtDNA content was positively correlated to the CCs mtDNA content [141]. A later study found that the number of copies of mtDNA in the CCs was predictive of the embryo quality during in vitro fertilization (IVF) cycles, with a positive predictive value of 84.4% and a negative predictive value of 82.1% [142]. This finding was confirmed in another study on 202 embryos that showed a significant correlation between the amount of mtDNA in the CCs of an oocyte and the morphokinetic quality of the resulting embryo [143]. Finally, a recent study of 84 COCs found that a significantly higher number of copies of mtDNA in the CCs of oocytes leading to implanted embryos compared to non-implanted embryos (Mean, respectively, 215 [sd 375] and 59 [sd 72], respectively, *p* < 0.0001) [144]. A multivariate analysis accounting for women’s age, embryo quality, and serum anti-mullerian hormone (AMH) level showed an independent relationship between the MtDNA content of the CCs and the potential of embryo implantation [144]. Future prospective studies are needed to confirm these findings.

### 4.2. Free mtDNA

The search for biomarkers has also led to the analysis of the embryo culture media. Embryos that reach the blastocyst stage have, in their culture media, a significantly higher mtDNA/Nuclear DNA ratio compared to embryos that stopped growing or had a slow growth [145]. Similarly, the amount of mtDNA found in the culture media could be correlated to certain morphokinetic parameters of the embryo [146]. Unfortunately, the risk of contamination of the culture media by exogenous DNA or mtDNA from the CCs around the embryo seems to be high, thus limiting the use of this biomarker [147].

### 4.3. MtDNA and Trophectoderm Biopsy

The mtDNA oocyte content has been positively correlated to its competence, but the predictive value of that content in embryo biopsies is yet to be established. Early studies on the subject showed that in the embryo at the cleavage state, as is the case for the oocyte, the mtDNA content was notably higher in good quality embryos [148], or in young patients compared to older patients (oocyte quality being related to maternal age) [149]. However, other studies failed to confirm these findings, with some even showing the opposite [125,150]. The discordant results can be explained by the large inter-embryo and inter-blastomere variability in the mtDNA content at a given stage [148]. For embryos at the blastocyst stage, a high mtDNA level in the trophectoderm seems to indicate poor embryo quality. Indeed, aneuploid blastocysts and those from older women have high mtDNA levels [149]. The implantation potential of a euploid embryo seems to be inversely correlated to the mtDNA content of trophectoderm cells. This finding was reported in several retrospective studies: Fragouli et al. in 2015 (131 blastocysts) [149], Diez-Juan et al. in 2015 (65 blastocysts) [125], Ravichandran et al. in 2017 (1505 blastocysts) [151], and later prospectively confirmed (199 blastocysts) [152]. It has been suggested that the amount of MtDNA in a trophectoderm biopsy reflects embryonic stress that could impact the implantation potential. Therefore, a high mtDNA level could mirror an abnormal mitochondrial activation because of energy requirements related to anomalies in embryo development [125]. This mechanism has already been confirmed in embryo carriers of pathogenic mutations in mtDNA. These embryos have significantly higher levels of mtDNA compared to controls, thus suggesting a compensatory replicative mechanism to increase the number of normal copies necessary for embryo development [153]. However, the aforementioned findings that “the lower the mtDNA content, the better” has been refuted by other authors, who did not find any difference between blastocysts, regardless of ploidy, woman’s age, or implantation potential [154,155,156]. Another study found that mtDNA quantitation did not distinguish between embryos that implanted and embryos that did not implant following a double embryo transfer (DET) [157]. In conclusion, the debate is still on [158,159], and the answer could be given by future studies that take into consideration the embryo culture conditions and the mtDNA quantification techniques on one hand, and the confounding factors that could impact the mtDNA content such as tobacco use or the body mass index (BMI) on the other [143,160].

## 5. Mitochondria as Targets to Improve Fertility

### 5.1. Improving the Mitochondrial Function

In recent years, worldwide studies have explored several methods to improve mitochondrial function by using pharmacologic agents capable of protecting from oxidative stress or increasing energy production (review in [161]). In animals, many agents have been proven efficient: coenzyme Q10, an alternative component of the mitochondrial respiratory chain with antioxidant properties, has been shown to decrease follicular atresia, improve the expression of mitochondrial genes in the oocyte, and restore mitochondrial function; rapamycine increased mitophagy and mitochondrial renewal, and resveratrol induced mitochondrial biogenesis and activity. In humans, rapamycine is only used as an immunosuppressant, but resveratrol and coenzyme Q10 are safe, and coenzyme Q10 is already widely used to counteract the effect of aging on female fertility. The activation of certain keys molecules, such as sirtuins (Sirt3), could be one of the best methods to improve mitochondrial biogenesis. Caloric restriction has also been proposed as a way to improve fertility. Studies in rodents have shown that decreasing the caloric intake by 40% significantly decreases mitochondrial damage and meiotic errors. In humans, a diet rich in proteins and low in sugars has been associated with an increase in the blastulation and pregnancy rate. To date, the use of antioxidants to improve fertility remains controversial, and a 2020 Cochrane meta-analysis failed to show a real beneficial impact in terms of pregnancy and live birth rates [162].

### 5.2. Cytoplasmic and Mitochondrial Transfer

#### 5.2.1. Mitochondrial Replacement Therapy

Mitochondrial replacement therapy (MRT) techniques have been developed in order to avoid the transmission of severe hereditary mitochondrial diseases caused by pathogenic mutations of mtDNA. There are several protocols for MRT: polar body transfer (PBT), maternal spindle transfer (MST), germinal vesicle transfer (GVT), and pronuclear transfer (PNT) (Tachibana, 2018; Greenfield, 2017, etc.). The first three techniques involve the transfer of the nuclear genome of the oocyte (or polar body) of a patient carrying a pathogenic MtDNA mutation into the cytoplasm of the oocyte of a donor. PNT involves the transfer of the pronuclei of the zygote of a woman carrying an mtDNA mutation into the cytoplasm of the zygote of a donor. MRT leads to a conceptus carrying both parental nuclear genomes and the mitochondrial genome of the donor, commonly referred to as the “three-parent baby”. This technique was first validated by the United Kingdom parliament in 2015 and later endorsed by HEFA (Human Fertilization and Embryology Authority) in 2016. The first baby conceived by MST was born in Mexico in 2016 [163]. However, these techniques remain open to debate, both for safety and ethical reasons. Indeed, there remains a concern that some amount of MtDNA from the recipient might persist, thus leading to a mitochondrial heteroplasmy (coexistence of two types of mitochondrial genomes), which in itself could be deleterious, or lead to the reemergence of the MtDNA carrying the pathogenic mutations during embryogenesis. Furthermore, there is a risk of disrupting the nuclear-cytoplasmic interaction, leading to major cellular dysfunction. Nevertheless, the development of these techniques has brought the focus back on the use of mitochondrial transfer for the treatment of several forms of infertility, most notably those due to ovarian aging or embryo cleavage arrest [164].

However, before these techniques become widely available, several studies are required to assess the short, middle, and long term risks on the offspring. The first meta-analyses on the subject have so far yielded conflicting results [165,166,167].

#### 5.2.2. Cytoplasmic or Mitochondrial Transfer for the Treatment of Infertility

The injection of a fraction of the cytoplasm of an oocyte from a young donor into the oocyte of an older recipient significantly improves the oocyte competence and allows for better embryo development. This method was used in the 2000s, especially in the United States, and led to around fifty live births [168]. However, the practice was banned by the FDA in 2002 because of several biological and ethical concerns.

There are several ways by which cytoplasmic transfer is believed to restore oocyte competence. Among different cytoplasmic factors potentially involved (RNAs, organelles, etc.), the mitochondria could be the most important. Indeed, the transfer of isolated mitochondria can, by itself, increase the ATP production of the oocyte, prevent oocyte apoptosis, and promote embryo development in several species, including humans [164]. However, as mentioned earlier, this technique carries the risk of mitochondrial heteroplasmy and the disruption of the nuclear-cytoplasmic interactions. In order to avoid that hazard, the transfer of isolated mitochondria deriving from somatic cells of the same person has been proposed. Unfortunately, somatic mitochondria have tissue-specific characteristics and seem to alter embryo development [169]. So far, the best results have been achieved using mitochondria derived from cells of ovarian origin. For instance, the transfer of mitochondria derived from follicular cells has given promising results in bovines and humans. However, it is known that oocyte atresia is triggered by apoptotic signals originating in the follicular cells, and there is therefore a risk of precipitating oocyte degeneration when transferring mitochondria from these cells. On the other hand, transferring somatic mitochondria from the same person does not solve the advanced age issue, as both the mitochondria and the recipient oocyte will have the same age. An alternative source could be autologous germ cells. Indeed, oogonial stem cells have been isolated in the ovaries of rodents and humans, even though their existence and accessibility is still debated by many. The injection of mitochondria derived from these stem cells for the treatment of infertility, referred to as AUGMENT (autologous germline mitochondrial energy transfer), has been authorized in some countries (Canada, Spain) and banned in others (USA). Results from early studies were encouraging [170], but a recent randomized trial was prematurely ended when intermediate analysis showed significantly lower blastulation rates in patients treated with this technique compared to controls (23% vs. 41%) [171]. It remains unclear whether the technique itself or the mitochondrial source was behind the negative outcome. In pigs, using competent oocytes as a source of mitochondrial transfer was shown to significantly improve oocyte competence [46]. In conclusion, and as has been recently highlighted [172], the ability of mitochondrial transfer to improve oocyte competence remains unclear, and further studies are required to confirm not only its efficiency, but also its safety.

## 6. Conclusions

The central role played by mitochondria in oocyte competence and embryo development has long been overlooked, but recent studies have focused on its importance, with sometimes contradictory results. However, researchers have found it difficult to pin down the exact role played by mitochondria in reproduction, mainly because, besides energy production, they are also centrally involved in the metabolism, cell signaling pathways, and gene expression regulation. This is why, to date, it has been challenging to find highly reliable and independent mitochondrial markers and to contemplate mitochondria as therapeutic targets. However, the field of mitochondrial research is continuously expanding with promising expectations.
